# Enriched environment ameliorates propagation of tau pathology and improves cognition in rat model of tauopathy

**DOI:** 10.3389/fnagi.2022.935973

**Published:** 2022-07-26

**Authors:** Veronika Mate, Tomas Smolek, Zuzana Vince Kazmerova, Santosh Jadhav, Veronika Brezovakova, Bernadeta Jurkanin, Ivana Uhrinova, Neha Basheer, Norbert Zilka, Stanislav Katina, Petr Novak

**Affiliations:** ^1^Institute of Neuroimmunology, Slovak Academy of Sciences, Bratislava, Slovakia; ^2^Axon Neuroscience R&D Services SE, Bratislava, Slovakia; ^3^Neuroimunology Institute, n.p.o., Bratislava, Slovakia; ^4^Institute of Mathematics and Statistics, Masaryk University, Brno, Czechia; ^5^Axon Neuroscience CRM Services SE, Bratislava, Slovakia

**Keywords:** histology, behavioral, enriched environment, propagation, seeding, tangle, tau

## Abstract

**Introduction:**

The typical symptoms of Alzheimer’s disease (AD) are cognitive impairment, disrupted spatial orientation, behavioral and psychiatric abnormalities, and later motor deficits. Neuropathologically, AD is characterized by deposits of pathological forms of endogenous proteins – amyloid-β, and neurofibrillary tau protein pathology. The latter closely correlates with brain atrophy and clinical impairment. Pharmacological therapies for these pathologies are largely absent, raising the question whether non-pharmacological interventions could be efficacious. Environmental factors can play a role in the manifestation of AD. It is unknown whether enriched environment (EE) can ameliorate the propagation of protein aggregates or their toxic components.

**Methods:**

We injected insoluble tau extracts from human brains with AD (600 or 900 ng per animal) into hippocampi of SHR72 transgenic rats that express non-mutated truncated human tau 151-391/4R, but usually do not develop hippocampal tangles. The rats had either standard housing, or could access an EE 5×/week for 3 months. Behavioral analysis included the Morris Water Maze (MWM). Histological analysis was used to assess the propagation of tau pathology.

**Results:**

Animals exposed to EE performed better in the MWM (spatial acquisition duration and total distance, probe test); unexposed animals improved over the course of acquisition trials, but their mean performance remained below that of the EE group. Enriched environment abrogated tau propagation and hippocampal tangle formation in the 600 ng group; in the 900 ng group, tangle formation was ∼10-fold of the 600 ng group, and unaffected by EE.

**Conclusion:**

Even a small difference in the amount of injected human AD tau can cause a pronounced difference in the number of resulting tangles. EE leads to a noticeably better spatial navigation performance of tau-injected animals. Furthermore, EE seems to be able to slow down tau pathology progression, indicating the possible utility of similar interventions in early stages of AD where tangle loads are still low.

## Introduction

Neurodegenerative diseases termed “tauopathies” are characterized by progressive accumulation of abnormal tau protein. The most prevalent tauopathy is Alzheimer’s disease (AD), where tau pathology initially manifests in the locus coeruleus and entorhinal cortex, and subsequently spreads in a stereotypical manner to other functionally connected brain areas (Franzmeier et al., 2020). Accumulated abnormal tau species spread across the brain *via* a “prion-like” mechanism involving cell-to-cell transmission of pathological tau moieties that induce template-mediated conformational change of physiological tau in affected cells (Brundin et al., 2010; Frost and Diamond, 2010; Jucker and Walker, 2013; Walker and Jucker, 2015; Mudher et al., 2017).

Increasing propagation and the amount of tau pathology correlate directly with cognitive deficits and brain atrophy both in severity and extent (Nelson et al., 2012; Walker and Jucker, 2015; Scholl et al., 2016), as well as predicting future brain tissue loss (La Joie et al., 2020). The speed of progression largely depends upon the amount of seeding-capable tau molecules (Aoyagi et al., 2019).

Internal factors, such as genetics and immune status play also a role in the onset of tau pathology. Similarly, external factors, such as stress, diet, or environment also play a crucial role (Dosunmu et al., 2007; Tanzi, 2012, 2013; Stephen et al., 2021). Conversely, enriched environments can increase the activity of neurons and cognitive abilities, and slow down cognitive decline (Li et al., 2017).

An experimental set up commonly employed to assess environmentally-induced benefits for cognitive function involves placing animals in large cages containing interesting objects such as toys, running wheels, rolls, nestlets, tunnels, and plastic tubes (Balthazar et al., 2018). Animals placed in such environment exhibit improvement in memory functions and decreased amounts of amyloid deposits (Fordyce and Wehner, 1993; Lazarov et al., 2005; Ziegler-Waldkirch et al., 2018; Balietti et al., 2021). Cognitive stimulation was also found to modulate kinase activity and tau phosphorylation, indicating possible relevance for tau pathology as well (Gerenu et al., 2013).

Given the numerous failures in the development of pharmacological interventions aimed at slowing or halting the progression of AD, increased attention is being given to non-pharmacological treatment and prevention approaches (Ngandu et al., 2015; Stephen et al., 2021). If efficacious, these could reduce the number of AD cases, help bridge over the time until a pharmacological treatment is available, and ultimately could be combined with the disease-modifying drugs for even greater effect.

In our study, we evaluated the effect of enriched environment on spreading of tau pathology. Pathology was induced *via* intra-hippocampal administration of sarcosyl-insoluble tau protein isolated from human brains with AD into a transgenic rat model of human tauopathy (Smolek et al., 2019a,b). We employed SHR72 transgenic rats expressing human non-mutated truncated tau protein aa151-391; these animals normally do not develop neurofibrillary tangle (NFT) pathology in the hippocampus (Zilka et al., 2006; Koson et al., 2008). Then, we exposed animals to environmental enrichment (EE) and evaluated its effect on cognition and tau spreading.

## Materials and methods

### Human brain samples

Human brain material was procured from the brain collection of the University of Geneva, Switzerland, in compliance with their material transfer agreement. The material (frozen, post-mortem delay 4 h) originated from a subject with AD (female, aged 87 years, Braak stage 5). Parietal cortex tissue was used for the isolation of the sarcosyl-insoluble tau fraction. The utilized material was the same as used in our previous study; see Smolek et al. (2019b) for Western blot results of the tau extract.

### Isolation of sarcosyl-insoluble tau from human Alzheimer’s disease brains

Sarcosyl-insoluble tau was extracted according to published protocols (Jadhav et al., 2015). Briefly, brain tissue was homogenized in a buffer containing 20 mM Tris, 0.8 M NaCl, 1 mM EGTA, 1 mM EDTA, and 10% sucrose (Sigma Aldrich, St. Louis, MO, United States, S7903-1KG) supplemented with protease inhibitors (Complete, EDTA free, Roche Diagnostics, United States) and phosphatase inhibitors (1 mM sodium orthovanadate, Sigma-Aldrich, St. Louis, MO, United States, S6508-50G; 20 mM sodium fluoride, Sigma-Aldrich, St. Louis, MO, United States, S7920-100G). After centrifugation at 20,000 × *g* for 20 min the supernatant (S1) was collected, and small fraction was saved as total protein extract; 40% w/v of *N*-lauroylsarcosine (sarcosyl) (Sigma Aldrich, St. Louis, MO, United States, L5777-50G) in water was added to a final concentration of 1% and mixed by stirring for 1 h at room temperature. Then sample was centrifuged at 100,000 × *g* for 1 h at 25°C using Beckmann TLA-100 (Beckmann Instrument Inc., Fullerton, CA, United States). Pellets (P2) were washed and re-suspended in PBS to 1/50 volume of S1 fraction and sonicated briefly.

Before the injection into hippocampus, human AD brain isolates were examined for presence of PHF on electron microscope and for presence of tau by Western blot analysis with pan-tau antibody DC25. These analyses confirmed the presence of PHFs and various high and low molecular weight tau species, respectively.

The semiquantitative estimation of sarcosyl-insoluble tau was performed as described previously (Smolek et al., 2019b). The intensities of the samples and tau 40 were quantified *via* densitometry using AIDA Biopackage (Advanced Image Data Analyzer software; Raytest, Germany). The concentration of insoluble tau protein was estimated using a standard curve with reference intensities of known concentrations of recombinant tau 2N4R (tau 40).

### Stereotaxic surgery

Rats were anesthetized through intraperitoneal injection of a cocktail containing Zoletil (30 mg/kg) (Virbac, Carros, France, 5 mL) and Xylariem (10 mg/kg) (Ecuphar N.V., Oostcamp, Bruges, Belgium, 50 mL). Animals were fixed to a stereotaxic apparatus (Kopf Instruments, Los Angeles, CA, United States). An UltraMicroPump III (UMP III) Micro-syringe injector and Micro4 Controller (World Precision Instruments, Sarasota, FL, United States) were used for application. Stereotaxic coordinates for the injection into hippocampus were A/P: −3.6 mm, L: ±2.0 mm, D/V: 3.3 mm from bregma (Paxinos and Watson, 1997); in line with our previous study (Smolek et al., 2019b).

### Animals

The study was performed on 2 months old transgenic male rats (line SHR72) expressing human truncated tau protein aa151-391/4R. These animals develop neurofibrillary pathology in the brainstem and spinal cord, but not in the hippocampus (Zilka et al., 2006; Koson et al., 2008). All animals were housed under standard laboratory conditions with free access to water and food and kept under diurnal lighting cycle. Animals were divided into three experimental groups:

1st Group: 600 ng AD PHF group – 26 transgenic animals, each injected bilaterally with 1.5 μL of 200 ng/μL of insoluble tau fraction; 600 ng of insoluble tau protein was injected per animal in total. Subsequently, animals were divided into three groups: *600 ng AD PHF Enriched* – exposed to enriched environment (*n* = 13) and *600 ng AD PHF Non-enriched* – sedentary control group (*n* = 13).

2nd Group: 900 ng AD PHF group – 20 animals were injected bilaterally with 1.5 μL of 300 ng/μL of insoluble tau fraction; 900 ng of insoluble tau protein was injected per animal in total. Subsequently, animals were divided into two groups: *900 ng AD PHF Enriched* – exposed to enriched environment (*n* = 10) and *900 ng AD PHF Non-enriched* – sedentary control group (*n* = 10).

3rd Group: PBS group – 20 animals were injected with 3 μL of PBS, bilaterally. Subsequently, animals were divided into two groups: *PBS enriched* – exposed to enriched environment (*n* = 10) and *PBS non-enriched* – sedentary control group (*n* = 10). PBS was deemed an appropriate control, as our previous research shows that the impact of injecting PBS and healthy brain extract is the same (Smolek et al., 2019b).

### Enriched environment

**FIGURE 1:**
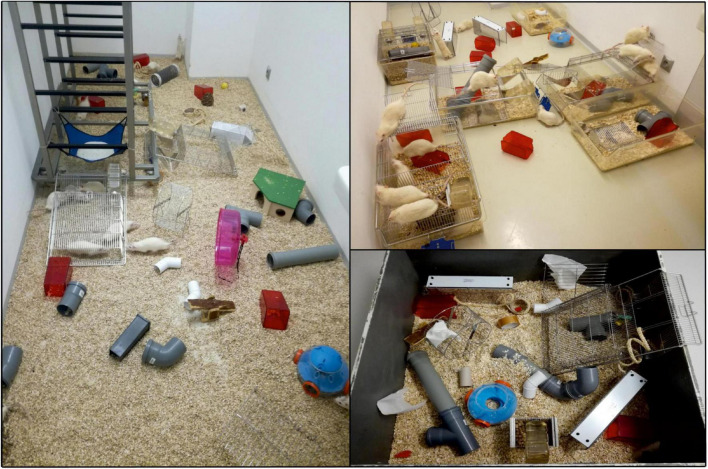
Illustration of enriched environment in laboratory conditions. Animals are introduced to novel objects, social housing, and exercise opportunities to maximize their exploration and exercise. Objects include running wheels, shelters, paper rolls, toys or different types of material to gnaw on, which typically vary in color, size or shape.

The animals in enriched group were all kept together to facilitate social interaction and enjoyed various novel stimuli, such as running wheels, shelters, paper rolls, toys and different types of material to gnaw on. The location of objects was regularly changed, and novel objects were made available every day. The size of the environment was 1.6 m × 3.8 m (6.08 m^2^). Rats were allowed to explore enriched environment daily for 6 h, 5 times per week (Figure 1), and were housed under conditions equivalent to the non-enriched control outside these hours.

Non-enriched (sedentary) groups were housed in standard plastic cages (555 mm × 345 mm × 195 mm), 4–5 animals per cage with food and water *ad libitum*. The rat colony and testing rooms were maintained in a temperature (22 ± 2°C) and humidity-controlled environment (45–65%) with a 12 h light/dark cycle. The experiments were concluded when the animals were aged 6 months. All experiments were performed in accordance with the Slovak and European Community Guidelines, with the approval of the State Veterinary and Food Administration of the Slovak Republic (Ro-4429/16-221c, Ro-1008/17-221) and the Institute’s Ethical Committee.

### Behavioral tests

Behavioral tests were performed at the age of 5 months. One week prior to the first testing, rats were handled to minimize stress and maximize validity of measured data.

#### Morris water maze test

Hippocampal-dependent spatial navigation and memory were evaluated *via* the Morris Water Maze (MWM) test, a widely used test for examining of spatial and reversal learning abilities and reference memory (Morris et al., 1982). The MWM is a black circular pool (180 cm in diameter, 75 cm high) filled approximately half-way with opaque water (22 ± 1°C). The maze is virtually divided into four equal quadrants (NE, NW, SE, and SW); the platform is located in the middle of one of the quadrants. The principle of the MWM task is that the animal must search for a hidden platform that is submerged below the water surface and placed in a fixed location; this platform is the only place in the pool where it can rest without having to swim. On the walls around the maze visual cues for navigation to the platform are located; four 60-W lights placed by the sides of the maze provide illumination. The swim parameters are recorded using a video camera located above the center of the testing arena, which is connected to a computer (placed in the next room), supported by a video tracking system (EthoVision XT 9, Noldus, Netherlands).

Spatial learning abilities of experimental animals were measured during the spatial learning acquisition phase of MWM test lasting 4 days. The Reference memory testing paradigm consisted of spatial learning acquisition phase and a probe test (Table 1).

**TABLE 1:** Design set-up of the Morris Water Maze (MWM) test.

Days	Trial	Number of trials/animal
**1–4**	Spatial learning acquisition	4
**5**	Probe test – reference memory	1
**6**	Spatial reversal learning acquisition	4
**7**	Probe test – reversal learning	1

The spatial learning acquisition phase consists of four trials per day on 4 subsequent days. During this stage, the platform is consistently localized in the NE quadrant. We used three start positions, each in quadrants other than the one containing the platform. Each animal is placed in the circuit of the pool facing the wall, and observed for 60 s or until it finds the platform. If the animal failed to find the platform within the allotted time, it was guided to the platform and left there for approximately 15 s, to perceive the distal cues and remember the location of the platform.

Twenty-four hours after the last acquisition, the probe test was administered. The platform was removed from the pool/hidden below the water surface and the animal released from a random location. The tracking system determined how much time the animal spent in each quadrant and information about reference memory was recorded. The analyzed variable was the time spent in the target quadrant.

On the 6th day, the spatial reversal learning phase of MWM was performed. The platform position was changed to the SW quadrant. Spatial reversal trials were administered in order to find out if the animal was capable to extinguish its initial learning of the platform’s position and to identify a direct path to the new platform position. On the 7th day the reversal probe trial was administered to evaluate spatial reversal learning abilities of experimental groups. The analyzed variable was the time spent in the new target quadrant.

### Immunohistochemistry

Rats were deeply anesthetized before being sacrificed and perfused transcardially with 1× phosphate buffered saline (1 × PBS). Brains were removed and fixed in 4% paraformaldehyde overnight, followed by permeabilization in sucrose solutions (15, 25, and 30% for 24 h each). Brains were frozen in in 2-methyl butane and stored on −80°C. Frozen brains were serially cut into sagittal 40 μm-thick sections using a cryomicrotome (Leica CM1850, Leica Biosystems). The sections were blocked with Aptum Section block (Aptum Biologics Ltd., Southampton, United Kingdom, cat. # APO471-500, 500 mL) followed by incubation with primary antibodies overnight at 4°C (Table 2). Sections were stained with biotin-conjugated secondary antibodies and developed using the Vectastain ABC Kit (Vector Laboratories, Newark, CA, United States). After mounting, sections were evaluated using Olympus BX51 microscope equipped with Olympus DP27 digital camera (Olympus microscope solutions).

**TABLE 2:** List of antibodies used for Immunohistochemical staining.

Antibody	Clonality	Dilution	Source
Anti-human phospho tau AT8 (pS202/pT205)	Mouse monoclonal	1:1000	Thermo Scientific (IL, United States), Cat. # MN1020
Anti-phospho tau pS214	Rabbit polyclonal	1:1000	Invitrogen (CA, United States), Cat. # 44-742G
Anti-tau DC39N1 (aa45-68)	Mouse monoclonal	1:100	Axon Neuroscience (Bratislava, Slovakia)
DC25 (aa347-353)	Mouse monoclonal	None*	Axon Neuroscience (Bratislava, Slovakia)

*Supernatant from cultured hybridoma cells was usd.

From the 600 ng experiment, 5 animals from the enriched group and 4 animals from the non-enriched group were randomly selected for immunohistochemical examination. In the subsequent 900 ng experiment, immunohistochemical analysis was done in 10 animals per group to improve the sample size.

For immunohistochemical staining, every 8th sagittal section was used (resulting distance of sections 320 μm); 10 sections were used for quantification, encompassing the tissue range 0.4–3.9 mm lateral from the medial line. Quantification of NFTs positive for anti-tau specific antibodies was performed manually. All tangles in the respective region of interest were counted by an observer blinded to the housing type of animals.

### Statistical analysis

Data processing and statistical analyses were performed in R programming environment version 4.1.2 (R Core Team, 2022). All alternative hypotheses were two-sided and statistical tests were performed at a significance level equal to 0.05. All empirical confidence intervals (CIs) are of Wald type, 95%, and two-sided.

Separately for each group (1st Group, 2nd Group, and 3rd Group, for NFL pathology only 1st Group and 2nd Group), the effect of enriched environment on

(1) Spatial learning acquisition and spatial reversal learning acquisition was analyzed by variables cumulative duration (s) and total distance (m).

(2) Reference memory acquisition (probe test) was analyzed by variable time spent in the target (NE) quadrant (s), and on spatial reversal learning acquisition (probe test) by variable time spent in the target (SW) quadrant (s),

(3) NFT pathology was analyzed by variable number of positive inclusions per slice.

The null hypothesis that the mean difference between non-enriched and enriched environment is equal to zero was tested against the alternative hypothesis that the mean difference between non-enriched and enriched environment is not equal to zero by two-sample Wald statistics (*t*-statistics) with Satterthwaite error degrees of freedom taken from the mixed-effect linear regression model (MELRM) (Pinheiro and Bates, 2006) calculated profile method with proportional weights [in (1) and (3)], and by two-sample Student *t*-statistics with Welch degrees of freedom (Welch, 1947) [in (2)]. The MELRM models were used in the following forms:

(A) Variable ∼ environment + day + group: day + (1| subject), for spatial learning acquisition in (1),

(B) Variable ∼ environment + trial + group: trial + (1| subject), for spatial reversal learning acquisition in (1),

(C) Variable ∼ environment + (1| subject),

where environment (non-enriched and enriched), day (1 to 4) and trial (quadrants NE-1, SE, NW, and NE-2) are the fixed main effects; group: day and group: trial are the interaction of the first order; and subject is the random effect (random intercept). Applying these MELRM on repeated observations (here 4 per day or trial), the repeated observations per animal are taken to account individually and thus the variability within and between animals is correctly estimated. The results are summarized numerically by mean difference (non-enriched minus enriched), lower and upper bound of 95% CIs for mean difference, *p*-value, and graphically by mean profiles with 95% CIs for means per each environment [in (1) and (2)] and boxplots [in (3)].

## Results

### Environmental enrichment improves spatial learning and reference memory

The MWM is one of the most valuable tools for measurement of hippocampally dependent spatial navigation/learning and reference memory in animals. In essence, animals have to learn how to find a hidden platform when started from different, random locations around the perimeter of tank (Vorhees and Williams, 2006). To achieve this task, the animals have to form a “spatial orientation map” in the brain by using distal cues to navigate a direct path to the platform. In our experimental set-up, platform was located into NE quadrant. During 4 days of training spatial learning was estimated by evaluation of cumulative duration (s) and total distance (m) necessary to reach the platform. To assess reference memory, probe trial was administered 24 h after the last acquisition day (day 5).

**FIGURE 2:**
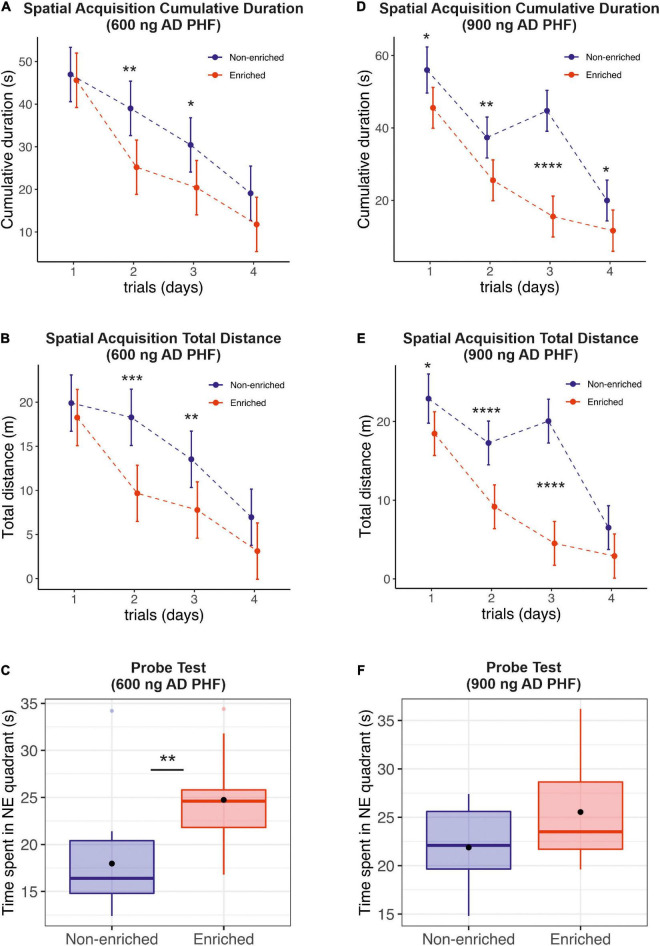
Evaluation of the effect of enriched environment on spatial learning and reference memory in transgenic rats injected with different doses of AD PHF. Cumulative duration **(A,D)**, total distance **(B,E)** and the time spent in platform quadrant (NE) during the 60-s probe trial **(C,F)** were measured, 1st group injected with 600 ng AD PHF **(A–C)**, 2nd group injected with 900 ng AD PHF **(D–F)**. Rats exposed to enriched environment needed less time to find the platform position **(A,D)** and traveled less total distance **(B,E)** than sedentary animals. Rats injected with lower dose (600 ng) of AD PHF and exposed to enriched environment spent significantly more time in platform quadrant than their non-enriched counterparts **(C)**. A similar trend was observed in the 2nd group of animals injected with higher dose of AD PHF, but the difference was not statistically significant. Graphs **(A,B,D,E)** represent mean and 95% of the mean of the respective experimental groups. Graphs **(C,E)** denote median, quartiles, with whiskers showing quartiles ±1.5× IQR. Dots denote arithmetic mean. *p < 0.05, **p < 0.01, ***p < 0.001, ****p < 0.0001.

Over the course of the spatial learning testing period, the performance of both groups, enriched and non-enriched, improved as evidenced by a decrease in cumulative duration (Figures 2A,D) and total distance (Figures 2B,E) needed to find the platform. The enriched group performed significantly better, especially during the second and third day of the spatial learning acquisition phase both in the experiment with 600 ng injection (Figures 2A,B; *p < 0.05, ^**^p < 0.01, ^***^p < 0.001, ^****^p < 0.0001, MELRM), and 900 ng injection of AD PHF (Figures 2D,E; ^**^p < 0.01, ^****^p < 0.0001, MELRM), both on total distance and cumulative duration, with maximum difference in favor of the enriched group being observed in the 900 ng experiment at day 3 (mean difference 29.19 s, *SD* 4.024) (see Supplementary Material for detailed listing of differences day-by-day). The beneficial effect of enriched environment on spatial learning was similar in rats injected with different doses of AD PHF, regardless of the level of NFT pathology in hippocampus. The sedentary group did not fully catch up to the enriched group, but the differences decreased at day 4, remaining significant only for cumulative duration in the 900 ng group (mean difference 8.32 s, 95% CI 0.31, 16.34).

In probe trials, sedentary animals in the 600 ng group spent significantly less time in the target quadrant compared to those exposed to enriched environment (mean difference −6.77 s, 95% CI −11.20, −2.34) (Figure 2C); in the 900 ng group, a similar but non-significant trend was observed in favor of the enriched group (mean difference −3.66 s, 95% CI −8.28, 0.96) (Figure 2E).

**FIGURE 3:**
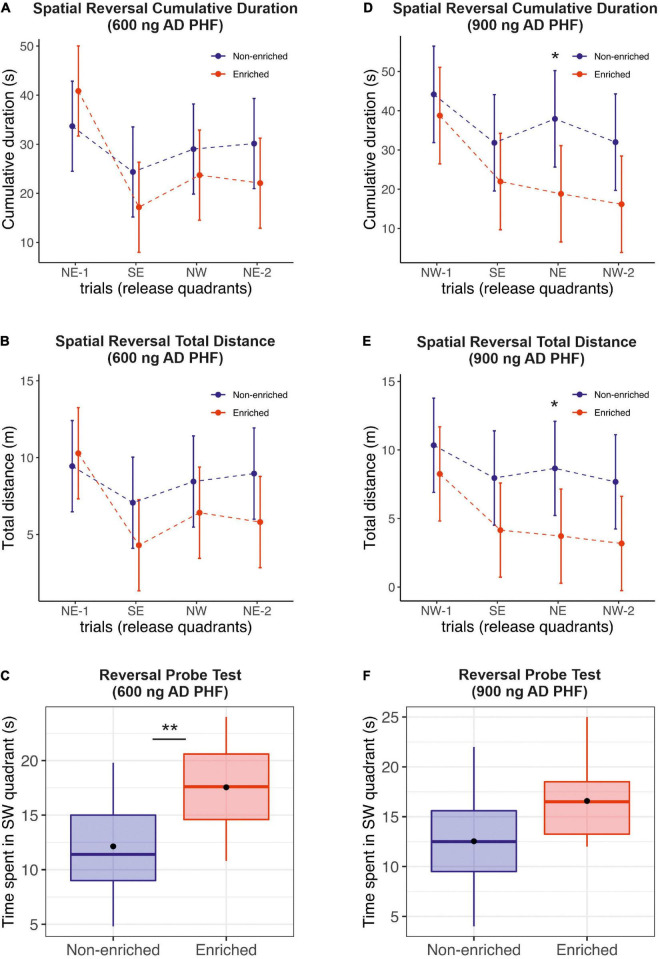
Evaluation of the effect of enriched environment on reversal learning in transgenic rats injected with different doses of AD PHF. Cumulative duration **(A,D)**, total distance **(B,E)** and the time spent in platform quadrant **(C,F)** during the reversal probe trial were measured, 1st group injected with 600 ng AD PHF **(A–C)**, 2nd group injected with 900 ng AD PHF **(D–F)**. Rats exposed to enriched environment generally required slightly less time **(A,D)** and less total distance **(B,E)** to find a new platform position (SW) compared to animals in non-enriched group (difference was not significant except at day 3 in the 900 ng group). Animals injected with lowed dose of AD PHF and exposed to enriched environment spent significantly more time in quadrant with new platform position (SW) than their non-enriched counterparts **(C)**. Animals injected with higher dose (900 ng) of AD PHF displayed a similar trend (*p* = 0.07). Graphs **(A,B,D,E)** represent means and 95% CIs of means for the respective experimental groups. Graphs **(C,E)** denote median, quartiles, with whiskers showing quartiles ±1.5× IQR. Dots denote arithmetic mean. *p < 0.05, **p < 0.01.

During spatial reversal acquisition (memory retention memory testing) animals exhibited spatial learning capabilities, and were able to memorize the new platform location in the SW quadrant. The performance of sedentary animals was slightly worse, with a significant difference observed for time and distance in the 900 ng group on day 3 (time: mean difference 19.1 s, 95% CI 1.72, 36.48; distance: mean difference 4.94 m, 95% CI 0.08, 9.80) (Figures 3A,B,D,E). See Supplementary Material for a detailed listing of day-by-day differences.

Similarly to the probe trial, in the reversal probe test, the enriched group in the 600 ng experiment performed significantly better than their sedentary counterparts (mean difference −5.42 s, 95% CI −8.84, −1.99; *p* = 0.0033). In the 900 ng experiment, we observed a similar trend in favor of the enriched group (mean difference −4.04 s, 95% CI −8.55, 0.47; *p* = 0.0756) (Figures 3C,F).

The experiment with PBS injection supports the above findings. Animals in the enriched group performed pronouncedly better in spatial acquisition both on cumulative duration and distance traveled, with differences significant at *p* = 0.0089 or better at all time points on days 1–4. Unlike their counterparts injected with AD PHF tau, these sham-injected animals did not show any significant difference between enriched and sedentary animals on the probe test and reversal probe test (see also Supplementary Figures 1C,F). In spatial reversal learning acquisition, some benefit of enrichment was initially still apparent, albeit with smaller differences than during spatial acquisition; e.g., in the second trial, the mean (SD) difference in time to locate the new platform was 26.44 (8.769) s (*p* = 0.0040), and total distance traveled differed by mean (SD) of 8.05 (2.223) m (*p* = 0.0007). By the fourth trial, the difference was minor, and no longer significant. See Supplementary Material for a detailed listing of day-by-day differences.

We have also evaluated the consistency of swimming speed between individual animals to exclude confounding of results e.g., by motor impairments. The distance traveled and time required to find the platform were closely intercorrelated, indicating fairly constant swimming speed and low variability in swimming speed between animals (600 ng: Spearman *r* = 0.9755, *p* < 0.0001; 900 ng: Spearman *r* = 0.9728, *p* < 0.0001). Absolute swimming speed of animals was similar between the dose groups, and within the expected range for healthy adult rats; mean (SD) speed was 0.33 (0.16) m/s in the 600 ng group, and 0.33 (0.13) m/s in the 900 ng group. Rats housed under non-enriched conditions swam faster than rats house in EE, though, with swim speeds of 0.38 (0.14) m/s and 0.29 (0.14) m/s, respectively (*p* < 0.0001), indicating that better cognition rather than better fitness was the reason for better MWM performance of the EE group. See Supplementary Material for correlation plots and group comparisons.

### Environmental enrichment significantly reduces the amount of tau pathology in the hippocampus of animals injected with the lower dose of Alzheimer’s disease PHF

In order to evaluate the impact of enriched environment on the propagation of tau pathology, we have induced progressive neurofibrillary pathology in the hippocampus of SHR72 transgenic rats as described previously (Smolek et al., 2019b) utilizing two different dosages of AD PHF tau (600 and 900 ng), and subsequently exposed the animals to environmental enrichment (enriched group) or standard housing conditions (sedentary group).

**FIGURE 4:**
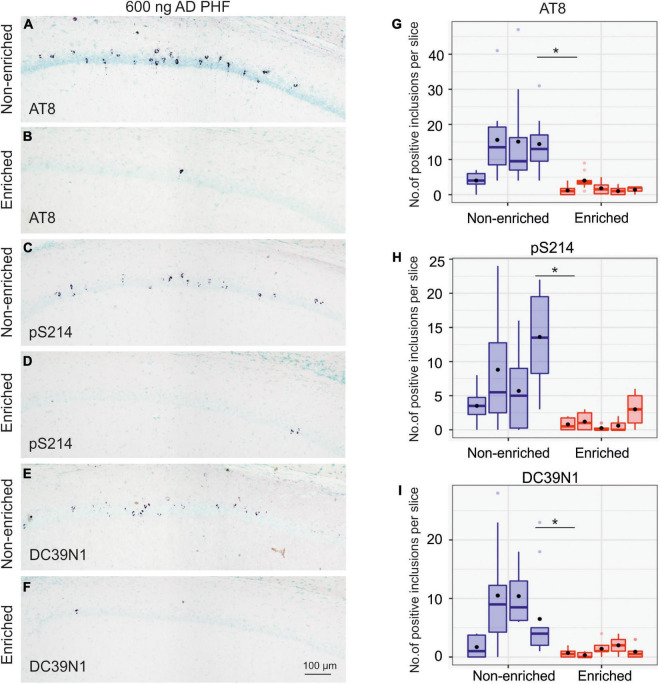
Enriched environment leads to less propagation of tau pathology in the group injected with 600 ng AD PHF tau. **(A–F)** Representative sagittal sections of the hippocampus of SHR72 rats injected with 600 ng human AD PHF tau. For IHC staining phospho-tau dependent antibodies AT8 **(A,B)**, pS214 **(C,D)** and human tau specific antibody DC39N1 were used **(E,F)**. Scale bar: 100 μm. **(G–I)** Quantitative analysis of tau pathology stained with phospho-tau dependent antibodies AT8 **(G)**, pS214 **(H)**, and human tau specific antibody DC39N1 **(I)**. A significant decrease in the amount of accumulated tau pathology is seen in the group exposed to enriched environment in comparison to the non-enriched, sedentary group (*p < 0.05; **p < 0.01). Each column denotes one animal. Boxes indicate median and quartiles for tangle counts per brain slice; whiskers indicate quartiles ±1.5× IQR. Black dots indicate arithmetic mean.

After 4 months of enriched environment, animals injected with 600 ng AD PHF tau displayed noticeably less neurofibrillary tangles than their sedentary (non-enriched) counterparts, with statistically significant differences for tangles labeled *via* each of the three antibodies used in the evaluation (AT8 *p* = 0.0043; pS214 *p* = 0.0121; DC39N1 *p* = 0.0122). See Figure 4 for representative hippocampal staining (A–F) and for quantitative comparisons (G–I).

Overall, animals injected with 900 ng developed pronouncedly more neurofibrillary tangles than those injected with 600 ng. Total AT8-positive tangle counts in the 600 ng non-enriched group ranged from 40 to 156 tangles, while those in the 900 ng non-enriched group ranged from 463 to 1,345 tangles.

**FIGURE 5:**
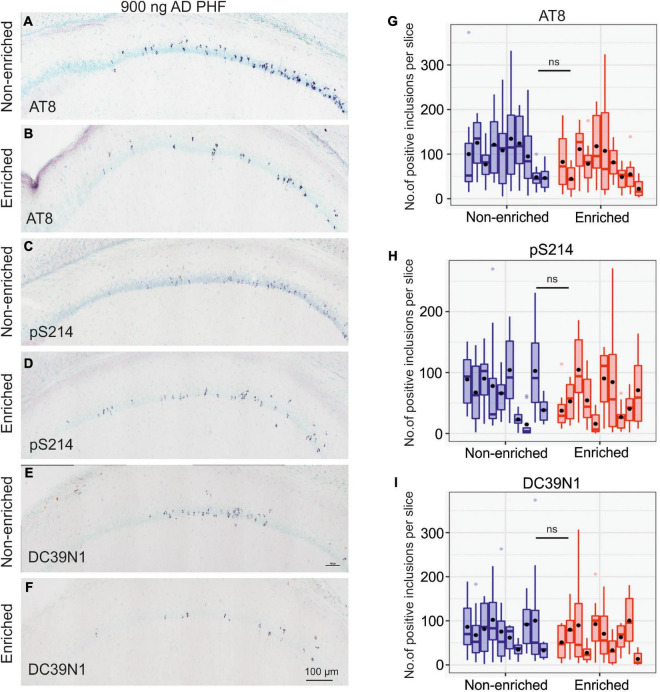
Enriched environment is insufficient to halt tau pathology propagation in the group injected with a high dose of 900 ng AD PHF tau. **(A–F)** Representative sagittal sections of the hippocampus of SHR72 rats injected with 900 ng human AD PHF tau. For IHC staining phospho-tau dependent antibodies AT8 **(A,B)**, pS214 **(C,D)** and human tau specific antibody DC39N1 were used **(E,F)**. Scale bar: 100 μm. **(G–I)** Quantitative analysis of tau pathology stained with phospho-tau dependent antibodies AT8 **(G)**, pS214 **(H)**, and human tau specific antibody DC39N1 **(I)**. No significant differences in the amount of accumulated tau pathology are seen between the groups (all *p* > 0.05). Each column denotes one animal. Boxes indicate median and quartiles for tangle counts per brain slice; whiskers indicate quartiles ±1.5× IQR. Black dots indicate arithmetic mean.

In the 900 ng experiment, the impact of enriched environment on tangle counts was far less pronounced; while the difference was still nominally in favor of the enriched group, it was not statistically significant (AT8 *p* = 0.1210; pS214 *p* = 0.4973; DC39N1 = 0.3689). See Figure 5 for representative hippocampal staining (A–F) and for quantitative comparisons (G–I).

## Discussion

The present study indicates a beneficial effect of environmental enrichment on learning and spatial navigation in a rat model of tau pathology; inhibition on the spread of tau pathology through environmental enrichment was seen for the lower of the two employed doses of AD tau.

Epidemiological studies suggests that cognitive stimulation and physical activity can prevent or delay the onset of human AD (Friedland et al., 2001; Wilson et al., 2002; Ngandu et al., 2015; Lopez-Ortiz et al., 2021). Previous research indicates the ability of EE to positively impact functional outcomes (Held et al., 1985), learning capacity and spatial memory of aged wild-type animals (Kobayashi et al., 2002; Montarolo et al., 2013). These studies also showed that the effect of long-term exposure to an EE starting at weaning was much greater than that of short-term exposure in aged rats, in line with the hypothesis that the effect of enrichment and exercise are “dose-dependent” (with time and intensity constituting the dose); similarly, the benefit of early intervention is highlighted. Current data indicate that enriched environment (EE) combines physical and intellectual stimulation and could have a beneficial effect on cognitive aspects in genetically modified animal models (Jankowsky et al., 2005; Lazarov et al., 2005; Wolf et al., 2006; Dong et al., 2007; Kazlauckas et al., 2011; Valero et al., 2011), as well as in humans (Yu et al., 2009; Ruthirakuhan et al., 2012; Ngandu et al., 2015). Even fairly short interventions combining cognitive stimulation and exercise can have a noticeable impact e.g., on psychomotor speed (Karssemeijer et al., 2019). The association of an active lifestyle and contributions of multimodal non-pharmacological interventions with better outcomes in Huntington’s disease is also documented (Novati et al., 2022).

Coincidentally, one of the first animal studies came from Huntington disease transgenic animals (van Dellen et al., 2000), and later ones from other neurodegenerative disorders such as Alzheimer’s disease or Parkinson’s disease (Laviola et al., 2008), supporting this notion. A positive impact of EE on motor functions was also observed in animals with motor deficits, specifically 6-OHDA lesioned rats (Urakawa et al., 2007) and tau-transgenic rats with progressive brainstem pathology (Stozicka et al., 2020). The latter study is especially relevant, as it shows that EE can ameliorate deficits caused by continuously accruing tau pathology, whereas EE in toxic lesion models is more akin to rehabilitation after one-time injury. Prolonged EE in a mouse model for familial AD (5xFAD) produced conflicting findings (Huttenrauch et al., 2017; Nakano et al., 2020) possibly because the model combines five familial mutations, each of which is sufficient to cause human disease with ∼100% penetrance.

A major limitation is that many *in vivo* studies are not sufficiently informative regarding the effect of enriched environment on pathophysiology, cognition and production of pathologically modified forms of AD proteins (Jankowsky et al., 2003, 2005; Lazarov et al., 2005). It is also important to note that many studies in the field were predominantly focused on modulation of Aβ levels using EE, but less is known about interaction between EE and tau protein. Neurofibrillary tangles composed of pathological tau species are an important pathological hallmark of AD, though, and seem closely tied to the phase of the disease where cognitive damage accrues (Novak et al., 1993; Goedert et al., 2006; Nelson et al., 2012). To date, the role of tau protein under environmentally enriched conditions has been not fully elucidated.

The model we have utilized may be uniquely suited to address this question. Specifically, the SHR72 line expresses human non-mutated truncated tau (aa151–391) protein and displays neurofibrillary tangle formation predominantly in brainstem (Zilka et al., 2006; Koson et al., 2008), with the hippocampus being normally spared. The human tau transgene expression provides a pre-conditioned medium that facilitates spreading of tau pathology while remaining faithful to what is observed in human AD by using human AD PHF tau extracts and non-mutant transgenic tau. Results from our previous *in vivo* experiments show that exogenous human AD tau is able to spread from the area of injection, as well as to induce and drive neurofibrillary pathology in this model (Smolek et al., 2019b). The main goal of this study was to test the hypothesis that EE could modulate cognitive functions and seeding ability of pathologically modified forms of tau protein (possibly *via* increasing neuronal activity) and ultimately affect propagation and spreading of tau pathology in the above model. After all, it was demonstrated that synaptic activation can promote clearance of tau oligomers by autophagosomes and lysosomes and can act as a protector for AD and other FTDs (Akwa et al., 2018), which aligns with the notion of low education and cognitive inactivity being a risk factor for AD.

In our study, a positive impact of EE on spatial navigation measured *via* the Morris Water Maze was observed, with better performance in spatial and reversal learning abilities and reference memory seen in both groups housed under enriched conditions, but especially in rats injected with the lower dose (600 ng) of AD PHF tau. The fact that non-enriched animals swam faster, but still needed more time and distance traveled to find the platform means that this difference was not caused merely by greater overall fitness of enriched animals, but rather is a true cognitive effect. In animals injected with 600 ng AD PHF tau, this improvement was echoed by a reduction in the amount of accumulated tau pathology. A possible explanation is that increased neuronal activity due to EE increased the amount of tau being secreted into the interstitial fluid (Yamada et al., 2014), whereupon it was taken up by activated microglia or peripheral macrophages and processed *via* cellular degradation systems (Majerova et al., 2014). Our previous study on the impacts of EE in the utilized SHR72 transgenic rats showed upregulated expression of microglial/macrophage markers Rt1-Ba, CD74, and Gpnmb, associated with increased number/overstimulation of microglia, or increased influx of blood-born monocytes into the brain. With Rt1-Ba and CD74 being responsible for antigen processing and increased phagocytic activity of microglia, and Gpnmb inhibiting certain activities of microglia/macrophages *via* negative regulation of pro-inflammatory molecules, this indicates a pronounced shift of microglial activity toward phagocytosis and protein clearance (Stozicka et al., 2020). Thus, modulation of immune function *via* EE (Singhal et al., 2014) could allow neurons to ‘outsource’ a part of the processing of pathological tau to immune cells.

The control experiment with PBS injection supports the findings from the groups injected with AD PHF tau. Specifically, it confirms the benefit of enriched environment even in these animals without hippocampal pathology.

Our findings tie in well with the notion that the speed of tau pathology progression is dependent on the quantity of seeding-capable tau molecules (Aoyagi et al., 2019), and that this relationship does not have to be linear. In fact, the body of evidence on tau spreading suggests that there are sub-threshold doses where spreading is prevented *via* homeostatic mechanisms, and presumably thresholds where production of pathological tau absolutely overwhelms the body’s attempts at homeostasis. In between lies the intervention window for various non-pharmacological approaches. Intervening early in the course of pathology is thus attractive from two standpoints. On one hand, slowing disease progression prior to the manifestation of pronounced cognitive loss and dementia is incomparably better for the patient than extending life at the dementia stage; on the other hand, the odds of success appear to be higher at disease stages where the extent and amount of neurofibrillary tau lesions is not insurmountable yet.

The protocol used in the present study has performed well, producing abundant numbers of neurofibrillary tangles at and beyond the injection site. To facilitate studies on propagation of tau pathology, and to promote comparability between experiments, it is necessary to advance and harmonize several aspects of methodology. Commonly, studies do not report the exact amount of pathological tau that was injected (Clavaguera et al., 2009); this issue can be addressed utilizing ELISA (Boluda et al., 2015), spectrophotometry (Nies et al., 2021), or Western blotting. It has to be noted that the seeding activity can pronouncedly (possibly 70-fold) differ between brain extracts from different tauopathy cases (Aoyagi et al., 2019). This said, tau loads necessary to produce pathology in different seeding studies were generally in the high nanogram to low microgram range (Boluda et al., 2015; Narasimhan et al., 2017; Albert et al., 2019; Nies et al., 2021). The study by Boluda et al. (2015) is of special interest, as the spreading pattern and quantity of pathology is evaluated across a wide range of tau doses, showing that with higher tau doses change both the extent and quantity of formed tau pathology. Ultimately, a measure of seeding units will be needed to guarantee comparability, very much like employed in prion research (Orru et al., 2017). An interim solution may be the preparation of large pooled seed material batches. As for the injection site, the hippocampus seems to be preferred across studies, due to its relevance to AD, abundant ipsi- and contralateral connections, and vulnerability of cells to tau pathology. The speed of propagation (and thus time until readout) is probably dependent on material and animal strain, with some groups reporting contralateral spreading already within a month (Boluda et al., 2015). To conclude, the concept of intervention *via* exercise and enrichment to counteract various aspects of neurodegeneration is well-supported by a range of animal and human studies; this study adds another stone to the mosaic, showing an impact of EE on tau pathology. What remains to be elucidated are the non-trivial details: optimal intervention protocols that maximize effort-to-benefit ratio; dosing (i.e., duration and intensity) of interventions; ideal intervention window; selection criteria for subjects where such intervention is meaningful; early therapy response readouts (Stephen et al., 2021). A simpler solution may be to enrich the environment of *everyone*, and see.

## Conclusion

Tau spreading and tangle formation occurs in a dose-dependent manner, with ∼10-fold differences occurring due to 50% difference in dose. Enriched environment improves spatial navigation of animals injected with AD PHF tau. With the lower dose of AD PHF tau, environmental enrichment is able to pronouncedly reduce neurofibrillary lesion formation, presumably *via* a mechanism involving modulation of microglial activity.

These findings highlight environmental enrichment as a viable approach to slow progression of tau protein pathology especially at early stages where overwhelming amounts of AD PHF tau haven’t formed yet.

## Limitations

The present study has some limitations which should be addressed in future research:

(i) Only a randomly selected subset of animals was analyzed histologically, leading to a small sample size for tangle quantification in the first animal cohort (600 ng).

(ii) Only two different dosages of AD PHF tau were used in the study (600 and 900 ng).

(iii) The study did not include evaluation of the brain stem nor evaluation of microglial markers.

(iv) The 600 ng, 900 ng, and PBS experiments utilized the same animal strain, same behavioral protocol, and same AD PHF tau extract for the 600 and 900 ng injection, but were conducted several months apart, reducing comparability somewhat.

(v) A more accurate quantification of tau pathology would be possible *via* a combination of tangle counting and quantitative biochemical analysis.

(vi) The design of the present study doesn’t allow the assessment of the contribution of individual components of the intervention (novel object enrichment, increased physical activity, increased social interaction, etc.) to the observed effect.

(vii) Comparability with other studies likely depends on the setup of the enriched environment, including environment dimensions.

(viii) While the MWM is a highly relevant cognitive test, expanding the assessment scope by adding a measure of anxiety, as well as another suitable behavioral test would improve the interpretability of results.

## Data availability statement

The raw data supporting the conclusions of this article will be made available by the authors, without undue reservation, to any qualified researcher.

## Ethics statement

All experiments were performed in accordance with the Slovak and European Community Guidelines, with the approval of the State Veterinary and Food Administration of the Slovak Republic (Ro-4429/16-221c and Ro-1008/17-221) and the Institute’s Ethical Committee.

## Author contributions

VM: design and conduct of behavioral studies, analysis of behavioral data, stereotactic surgery, and sampling. TS: sample processing, immunohistochemistry, analysis of IHC data, quantification of inclusions, and manuscript writing. ZK: data review and quality control, data management, and review of manuscript of intellectual content. SJ and VB: isolation of PHF proteins, protein analysis and quantification, and western blotting. BJ and IU: behavioral assessment (assistance). NB: manuscript writing and literature review. NZ: study supervision and study design. SK: principal statistician and manuscript writing. PN: manuscript writing and contribution to statistical analysis. All authors contributed to the article and approved the submitted version.
