# Supplementary material for: Enriched environment ameliorates propagation of tau pathology and improves cognition in rat model of tauopathy
Source: Front Aging Neurosci. 2022 Jul 26;14:935973. doi: 10.3389/fnagi.2022.935973 (PMC9363241; doi:10.3389/fnagi.2022.935973)
Supplement: Supplementary file 1 [file Data_Sheet_1.DOCX]

**Supplementary material** for:

*“Enriched environment ameliorates propagation of tau pathology and improves cognition in rat model of tauopathy”*

2022-07-06

Contents

[Results for the group injected with PBS buffer 2](#_Toc108016286)

[Detailed statistical results 3](#_Toc108016287)

[Association between duration and distance travelled to reach the platform 6](#_Toc108016288)

[Comparison of swimming speeds between groups 8](#_Toc108016289)

# Results for the group injected with PBS buffer


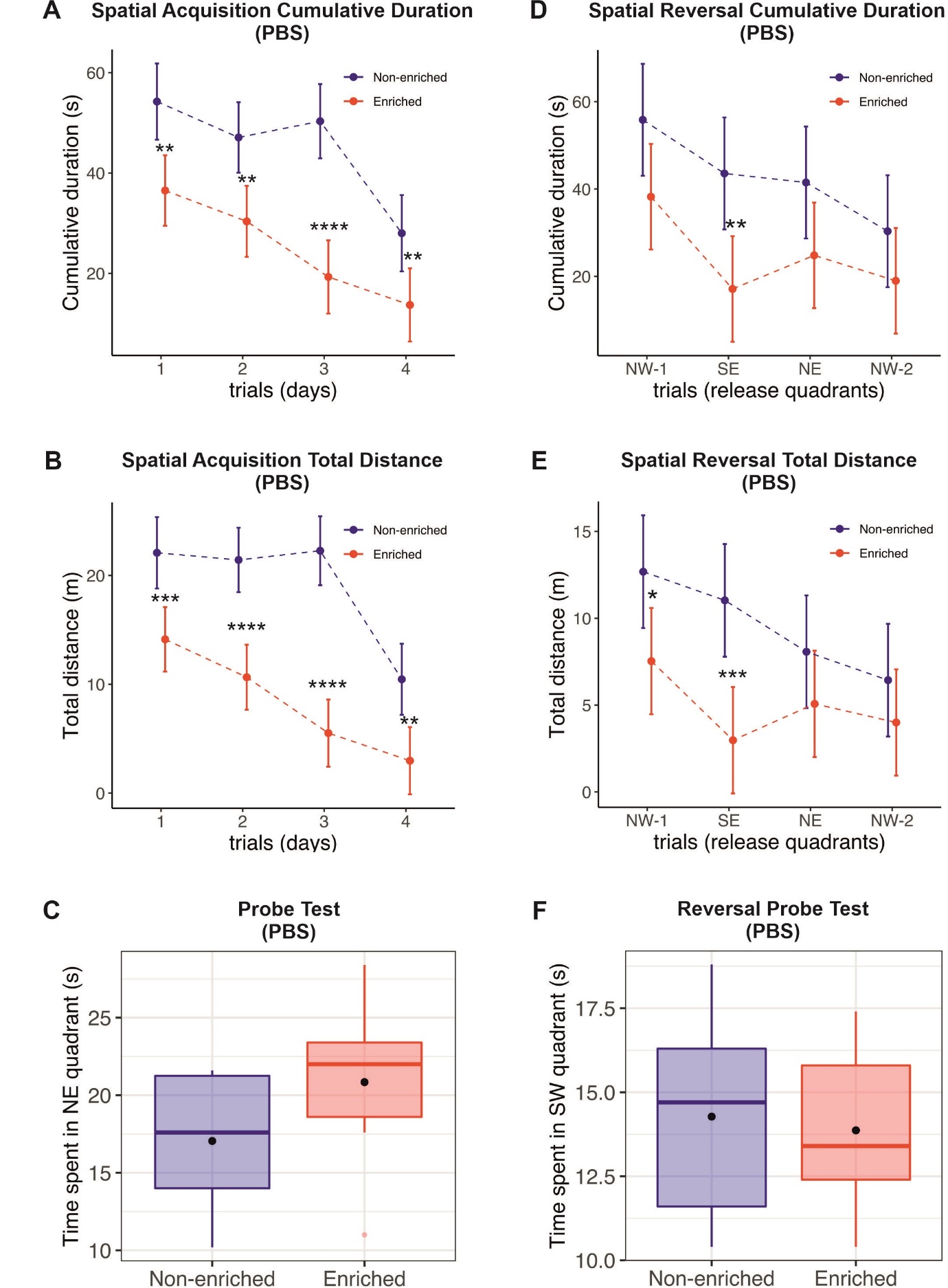


**Supplementary Figure 1: Animals housed in the enriched environment perform substantially better especially in the spatial acquisition phase of the Morris Water Maze.**

A, B, D, E: graphs represent mean ± 95% CI of the mean. C, F: Graphs denote median, quartiles, with whiskers showing quartiles ±1.5x IQR. Dots denote arithmetic mean. * = p < 0.05; ** = p < 0.01, *** = p < 0.001, **** = p < 0.0001

# Detailed statistical results

The most salient findings are described in the main manuscript. This section details statistical results day-by-day, variable by variable.

**Supplementary Table 1: MELRM results for group differences – Spatial acquisition**

| figure | description | variable | contrasts | mean diff | sd diff | p-value | 95% CI lower | 95% CI upper |
| --- | --- | --- | --- | --- | --- | --- | --- | --- |
| F02 A | Spatial Acquisition Cumulative duration (600 ng AD PHF) | Cumulative duration (s) | day 1: Non-enriched - Enriched | 1.4 | 4.52 | 0.7634 | -7.66 | 10.39 |
| F02 A | Spatial Acquisition Cumulative duration (600 ng AD PHF) | Cumulative duration (s) | day 2: Non-enriched - Enriched | 13.8 | 4.52 | 0.0033 | 4.79 | 22.84 |
| F02 A | Spatial Acquisition Cumulative duration (600 ng AD PHF) | Cumulative duration (s) | day 3: Non-enriched - Enriched | 10.0 | 4.52 | 0.0301 | 1.00 | 19.05 |
| F02 A | Spatial Acquisition Cumulative duration (600 ng AD PHF) | Cumulative duration (s) | day 4: Non-enriched - Enriched | 7.3 | 4.52 | 0.1109 | -1.72 | 16.33 |
| F02 B | Spatial Acquisition Total Distance (600 ng AD PHF) | Total distance (m) | day 1: Non-enriched - Enriched | 1.6 | 2.26 | 0.4697 | -2.87 | 6.16 |
| F02 B | Spatial Acquisition Total Distance (600 ng AD PHF) | Total distance (m) | day 2: Non-enriched - Enriched | 8.6 | 2.26 | 0.0003 | 4.10 | 13.13 |
| F02 B | Spatial Acquisition Total Distance (600 ng AD PHF) | Total distance (m) | day 3: Non-enriched - Enriched | 5.7 | 2.26 | 0.0134 | 1.23 | 10.27 |
| F02 B | Spatial Acquisition Total Distance (600 ng AD PHF) | Total distance (m) | day 4: Non-enriched - Enriched | 3.8 | 2.26 | 0.0942 | -0.68 | 8.35 |
| F02 D | Spatial Acquisition Cumulative Duration (900 ng AD PHF) | Cumulative duration (s) | day 1: Non-enriched - Enriched | 10.4 | 4.30 | 0.0166 | 1.93 | 18.94 |
| F02 D | Spatial Acquisition Cumulative Duration (900 ng AD PHF) | Cumulative duration (s) | day 2: Non-enriched - Enriched | 11.8 | 4.02 | 0.0041 | 3.83 | 19.79 |
| F02 D | Spatial Acquisition Cumulative Duration (900 ng AD PHF) | Cumulative duration (s) | day 3: Non-enriched - Enriched | 29.2 | 4.02 | 0.0000 | 21.21 | 37.17 |
| F02 D | Spatial Acquisition Cumulative Duration (900 ng AD PHF) | Cumulative duration (s) | day 4: Non-enriched - Enriched | 8.3 | 4.05 | 0.0420 | 0.31 | 16.34 |
| F02 E | Spatial Acquisition Total Distance (900 ng AD PHF) | Total distance (m) | day 1: Non-enriched - Enriched | 4.4 | 2.11 | 0.0375 | 0.26 | 8.63 |
| F02 E | Spatial Acquisition Total Distance (900 ng AD PHF) | Total distance (m) | day 2: Non-enriched - Enriched | 8.1 | 1.99 | 0.0001 | 4.17 | 12.05 |
| F02 E | Spatial Acquisition Total Distance (900 ng AD PHF) | Total distance (m) | day 3: Non-enriched - Enriched | 15.5 | 1.99 | 0.0000 | 11.61 | 19.48 |
| F02 E | Spatial Acquisition Total Distance (900 ng AD PHF) | Total distance (m) | day 4: Non-enriched - Enriched | 3.6 | 2.00 | 0.0732 | -0.35 | 7.57 |
| SUPPL A | Spatial Acquisition Cumulative duration (PBS) | Cumulative duration (s) | day 1: Non-enriched - Enriched | 17.7 | 5.16 | 0.0012 | 7.36 | 28.11 |
| SUPPL A | Spatial Acquisition Cumulative duration (PBS) | Cumulative duration (s) | day 2: Non-enriched - Enriched | 16.7 | 4.96 | 0.0016 | 6.73 | 26.73 |
| SUPPL A | Spatial Acquisition Cumulative duration (PBS) | Cumulative duration (s) | day 3: Non-enriched - Enriched | 31.0 | 5.18 | 0.0000 | 20.63 | 41.46 |
| SUPPL A | Spatial Acquisition Cumulative duration (PBS) | Cumulative duration (s) | day 4: Non-enriched - Enriched | 14.3 | 5.26 | 0.0089 | 3.74 | 24.85 |
| SUPPL B | Spatial Acquisition Total Distance (PBS) | Total distance (m) | day 1: Non-enriched - Enriched | 8.0 | 2.22 | 0.0006 | 3.54 | 12.37 |
| SUPPL B | Spatial Acquisition Total Distance (PBS) | Total distance (m) | day 2: Non-enriched - Enriched | 10.8 | 2.11 | 0.0000 | 6.57 | 14.98 |
| SUPPL B | Spatial Acquisition Total Distance (PBS) | Total distance (m) | day 3: Non-enriched - Enriched | 16.8 | 2.22 | 0.0000 | 12.34 | 21.17 |
| SUPPL B | Spatial Acquisition Total Distance (PBS) | Total distance (m) | day 4: Non-enriched - Enriched | 7.5 | 2.26 | 0.0014 | 3.00 | 11.97 |
| F03 A | Spatial Reversal Cumulative duration (600 ng AD PHF) | Cumulative duration (s) | trial NE-1: Non-enriched - Enriched | -7.2 | 6.54 | 0.2756 | -20.15 | 5.81 |
| F03 A | Spatial Reversal Cumulative duration (600 ng AD PHF) | Cumulative duration (s) | trial SE: Non-enriched - Enriched | 7.2 | 6.54 | 0.2746 | -5.80 | 20.16 |
| F03 A | Spatial Reversal Cumulative duration (600 ng AD PHF) | Cumulative duration (s) | trial NW: Non-enriched - Enriched | 5.3 | 6.54 | 0.4189 | -7.67 | 18.29 |
| F03 A | Spatial Reversal Cumulative duration (600 ng AD PHF) | Cumulative duration (s) | trial NE-2: Non-enriched - Enriched | 8.1 | 6.54 | 0.2206 | -4.92 | 21.04 |
| F03 B | Spatial Reversal Total Distance (600 ng AD PHF) | Total distance (m) | trial NE-1: Non-enriched - Enriched | -0.8 | 2.11 | 0.6921 | -5.04 | 3.36 |
| F03 B | Spatial Reversal Total Distance (600 ng AD PHF) | Total distance (m) | trial SE: Non-enriched - Enriched | 2.8 | 2.11 | 0.1938 | -1.43 | 6.96 |
| F03 B | Spatial Reversal Total Distance (600 ng AD PHF) | Total distance (m) | trial NW: Non-enriched - Enriched | 2.0 | 2.11 | 0.3403 | -2.17 | 6.22 |
| F03 B | Spatial Reversal Total Distance (600 ng AD PHF) | Total distance (m) | trial NE-2: Non-enriched - Enriched | 3.2 | 2.11 | 0.1392 | -1.04 | 7.35 |
| F03 D | Spatial Reversal Cumulative duration (900 ng AD PHF) | Cumulative duration (s) | trial NW-1: Non-enriched - Enriched | 5.4 | 8.70 | 0.5371 | -11.98 | 22.78 |
| F03 D | Spatial Reversal Cumulative duration (900 ng AD PHF) | Cumulative duration (s) | trial SE: Non-enriched - Enriched | 9.9 | 8.70 | 0.2604 | -7.50 | 27.26 |
| F03 D | Spatial Reversal Cumulative duration (900 ng AD PHF) | Cumulative duration (s) | trial NE: Non-enriched - Enriched | 19.1 | 8.70 | 0.0317 | 1.72 | 36.48 |
| F03 D | Spatial Reversal Cumulative duration (900 ng AD PHF) | Cumulative duration (s) | trial NW-2: Non-enriched - Enriched | 15.8 | 8.70 | 0.0736 | -1.56 | 33.20 |
| F03 E | Spatial Reversal Total Distance (900 ng AD PHF) | Total distance(m) | trial NW-1: Non-enriched - Enriched | 2.1 | 2.43 | 0.3931 | -2.77 | 6.95 |
| F03 E | Spatial Reversal Total Distance (900 ng AD PHF) | Total distance(m) | trial SE: Non-enriched - Enriched | 3.8 | 2.43 | 0.1232 | -1.06 | 8.66 |
| F03 E | Spatial Reversal Total Distance (900 ng AD PHF) | Total distance(m) | trial NE: Non-enriched - Enriched | 4.9 | 2.43 | 0.0465 | 0.08 | 9.80 |
| F03 E | Spatial Reversal Total Distance (900 ng AD PHF) | Total distance(m) | trial NW-2: Non-enriched - Enriched | 4.5 | 2.43 | 0.0691 | -0.36 | 9.36 |
| SUPPL D | Spatial Reversal Cumulative duration (PBS) | Cumulative duration (s) | trial NW-1: Non-enriched - Enriched | 17.6 | 8.77 | 0.0500 | 0.00 | 35.21 |
| SUPPL D | Spatial Reversal Cumulative duration (PBS) | Cumulative duration (s) | trial SE: Non-enriched - Enriched | 26.4 | 8.77 | 0.0040 | 8.83 | 44.05 |
| SUPPL D | Spatial Reversal Cumulative duration (PBS) | Cumulative duration (s) | trial NE: Non-enriched - Enriched | 16.7 | 8.77 | 0.0629 | -0.93 | 34.29 |
| SUPPL D | Spatial Reversal Cumulative duration (PBS) | Cumulative duration (s) | trial NW-2: Non-enriched - Enriched | 11.4 | 8.77 | 0.2015 | -6.26 | 28.96 |
| SUPPL E | Spatial Reversal Total Distance (PBS) | Total distance (m) | trial NW-1: Non-enriched - Enriched | 5.1 | 2.22 | 0.0245 | 0.69 | 9.61 |
| SUPPL E | Spatial Reversal Total Distance (PBS) | Total distance (m) | trial SE: Non-enriched - Enriched | 8.1 | 2.22 | 0.0007 | 3.59 | 12.51 |
| SUPPL E | Spatial Reversal Total Distance (PBS) | Total distance (m) | trial NE: Non-enriched - Enriched | 3.0 | 2.22 | 0.1829 | -1.46 | 7.46 |
| SUPPL E | Spatial Reversal Total Distance (PBS) | Total distance (m) | trial NW-2: Non-enriched - Enriched | 2.4 | 2.22 | 0.2782 | -2.02 | 6.90 |

All results obtained using: result ~ group + day + group:day + (1|subject), or result ~ group + trial + group:trial + (1|subject), respectively. P-values are colour-coded, with green fields indicating better significance. P-values < 0.0001 are shown as 0.0000.

**Supplementary Table 2: MELRM results for group differences – tangle counts**

| **figure** | **description** | **variable** | **contrasts** | **mean diff** | **sd diff** | **p-value** | **95% CI lower** | **95% CI upper** |
| --- | --- | --- | --- | --- | --- | --- | --- | --- |
| F04 G | AT8 | No. of positive inclusions per slice | Non-enriched - Enriched | 10.4 | 2.51 | 0.0043 | 4.46 | 16.33 |
| F04 H | pS214 | No. of positive inclusions per slice | Non-enriched - Enriched | 6.7 | 2.01 | 0.0121 | 1.98 | 11.48 |
| F04 I | DC39N1 | No. of positive inclusions per slice | Non-enriched - Enriched | 6.2 | 1.86 | 0.0123 | 1.83 | 10.60 |
| F05 G | AT8 | No. of positive inclusions per slice | Non-enriched - Enriched | 23.1 | 14.19 | 0.121 | -6.72 | 52.92 |
| F05 H | pS214 | No. of positive inclusions per slice | Non-enriched - Enriched | 9.5 | 13.68 | 0.4973 | -19.27 | 38.23 |
| F05 I | DC39N1 | No. of positive inclusions per slice | Non-enriched - Enriched | 11.3 | 12.25 | 0.3689 | -14.45 | 37.03 |

All results obtained using: result ~ group + (1|subject). P-values are colour-coded, with green fields indicating greater significance.

**Supplementary Table 3: results for group differences – probe test**

| **figure** | **description** | **variable** | **contrasts** | **mean diff** | **p-value** | **95% CI lower** | **95% CI upper** |
| --- | --- | --- | --- | --- | --- | --- | --- |
| F02 C | Probe Test (600 ng AD PHF) | Time spent in NE quadrant (s) | Non-enriched - Enriched | -6.8 | 0.0043 | -11.2 | -2.34 |
| F02 F | Probe test (900ng AD PHF) | Time spent in NE quadrant (s) | Non-enriched - Enriched | -3.7 | 0.113 | -8.279 | 0.9593 |
| F03 C | Reversal Probe Test (600 ng AD PHF) | Time spent in SW quadrant (s) | Non-enriched - Enriched | -5.4 | 0.0033 | -8.843 | -1.988 |
| F03 F | Reversal Probe Test (900 ng AD PHF) | Time spent in SW quadrant (s) | Non-enriched - Enriched | -4.0 | 0.0756 | -8.546 | 0.4657 |
| SUPPL C | Probe Test (PBS) | Time spent in NE quadrant(s) | Non-enriched - Enriched | -3.8 | 0.1203 | -8.705 | 1.1159 |
| SUPPL F | Reversal Probe Test (PBS) | Time spent in SW quadrant(s) | Non-enriched - Enriched | 0.4 | 0.7632 | -2.453 | 3.2692 |

All results obtained using a t-test. P-values are colour-coded, with green fields indicating greater significance.

# Association between duration and distance travelled to reach the platform

Test of zero Spearman correlation where p-value is calculated exactly using the permutation approach were used to assess the relationship between duration and distance travelled to reach the platform (Graphpad Prism, v. 8.4.3). Test statistic is not used in this approach. Null hypothesis: Spearman correlation coefficient is equal to zero vs alternative hypothesis: Spearman correlation coefficient is not equal to zero (two-sided alternative hypothesis).

**Supplementary Figure 2: Distance travelled and duration to find the platform are highly inter-correlated (600 ng group).**

Linear regression line with 95% CI shown. Four trials per animal over the course of four days are shown.

Spearman r = 0.9755; 95% CI 0.9702 to 0.9798; p <0.0001

**Supplementary Figure 3: Distance travelled and duration to find the platform are highly inter-correlated (900 ng group).**

Linear regression line with 95% CI shown. Four trials per animal over the course of four days are shown.

Spearman r = 0.9728; 95% CI 0.9658 to 0.9783; P (two-tailed) <0.0001

# Comparison of swimming speeds between groups

**Supplementary Figure 4: Animals housed under non-enriched conditions swim faster than their counterparts housed under enriched conditions.**

Regardless of faster swim speed, non-enriched animals take longer and have to travel further to reach the platform. The faster swim speed could be attributable to anxiety. Line and error show median and quartiles. Each dot represents one trial (i.e., 15-16 trials per animal). Two outlier trials are not shown (0.75 m/s and 1.5 m/s, respectively), but are included in the analysis.

The null hypothesis that the mean difference in swimming speed between non-enriched and enriched environment is equal to zero was tested against the alternative hypothesis that the mean difference between non‑enriched and enriched environment is not equal to zero by two-sample Student t‑statistics with Welch degrees of freedom.

**600 ng**: Two-sample t test with Welch's correction. Mean difference 0.07805 m/s, 95% CI 0.04903 to 0.1071, p-value <0.0001, Welch-corrected t=5.287, df=408.6.

**900 ng**: Two-sample t test with Welch's correction. Mean difference 0.09811 m/s, 95% CI 0.07084 to 0.1254, p-value <0.0001, Welch-corrected t=7.080, df=304.2.

**Supplementary Figure 5: Animals housed under non-enriched conditions swim faster than their counterparts housed under enriched conditions (data breakdown by animal).**

Regardless of faster swim speed, non-enriched animals take longer and have to travel further to reach the platform. The faster swim speed could be attributable to anxiety. Line and error show median and quartiles. Each dot represents one trial (i.e., 15-16 trials per animal). Two outlier trials are not shown (0.75 m/s and 1.5 m/s, respectively), but are included in the analysis.
